# Antibacterial and Anti-Biofilm Activities of Essential Oil Compounds against New Delhi Metallo-β-Lactamase-1-Producing Uropathogenic *Klebsiella pneumoniae* Strains

**DOI:** 10.3390/antibiotics11020147

**Published:** 2022-01-24

**Authors:** Paweł Kwiatkowski, Monika Sienkiewicz, Agata Pruss, Łukasz Łopusiewicz, Nikola Arszyńska, Iwona Wojciechowska-Koszko, Anna Kilanowicz, Barbara Kot, Barbara Dołęgowska

**Affiliations:** 1 Department of Diagnostic Immunology, Pomeranian Medical University in Szczecin, Powstancow Wielkopolskich 72, 70-111 Szczecin, Poland; pawel.kwiatkowski@pum.edu.pl (P.K.); arszynska00@gmail.com (N.A.); iwona.koszko@pum.edu.pl (I.W.-K.); 2 Department of Pharmaceutical Microbiology and Microbiological Diagnostic, Medical University of Lodz, Muszynskiego St. 1, 90-151 Lodz, Poland; 3 Department of Laboratory Medicine, Pomeranian Medical University in Szczecin, Powstancow Wielkopolskich 72, 70-111 Szczecin, Poland; agata.pruss@pum.edu.pl (A.P.); barbara.dolegowska@pum.edu.pl (B.D.); 4 Center of Bioimmobilisation and Innovative Packaging Materials, Faculty of Food Sciences and Fisheries, West Pomeranian University of Technology Szczecin, Janickiego 35, 71-270 Szczecin, Poland; lukasz.lopusiewicz@zut.edu.pl; 5 Department of Toxicology, Faculty of Pharmacy, Medical University of Lodz, 90-151 Lodz, Poland; anna.kilanowicz@umed.lodz.pl; 6 Institute of Biological Sciences, Faculty of Exact and Natural Sciences, Siedlce University of Natural Sciences and Humanities, 14 Bolesława Prusa Str., 08-110 Siedlce, Poland; barbara.kot@uph.edu.pl

**Keywords:** uropathogenes, essential oil compounds, biofilm biomass reduction, *Klebsiella pneumoniae* NDM

## Abstract

The World Health Organization points out that the opportunistic pathogen *Klebsiella pneumoniae* that causes various infections among others, urinary tract infections (UTIs), is one of the high-priority species due to a global problem of antimicrobial resistance. The aim of this study was to investigate antibacterial and anti-biofilm activities of chosen constituents of essential oils against NDM-1-producing, uropathogenic *K. pneumoniae* strains. The genes encoding lipopolysaccharide (*uge*, *wabG*), adhesin gene *fimH* (type I fimbriae) and gene encoding carbapenemase (*bla*_NDM-1_) for all tested strains were detected by PCR amplification. The *K. pneumoniae* ATCC BAA-2473 reference strain was *uge*- and *bla*_NDM-1_-positive. The effectiveness of fifteen essential oil compounds (EOCs) (linalool, β-citronellol, linalyl acetate, menthone, (−)-menthol, (+)-menthol, geraniol, eugenol, thymol, *trans*-anethole, farnesol, β-caryophyllene, (R)-(+)-limonene, 1,8-cineole, and carvacrol) was assessed by determining the MIC, MBC, MBC/MIC ratio against *K. pneumoniae* strains by the microdilution method. Anti-biofilm properties of these compounds were also investigated. Thymol, carvacrol and geraniol exhibited the best antibacterial and anti-biofilm activities against uropathogenic NDM-1-producing *K. pneumoniae* isolates. Results of our investigations provide a basis for more detailed studies of these phytochemicals on their application against uropathogenic *K. pneumoniae*.

## 1. Introduction

*Klebsiella pneumoniae* is an opportunistic pathogen that causes various infections, mainly respiratory, wound, bloodstream and urinary tract infections (UTIs) [1]. *K. pneumoniae* is considered an important uropathogen in both hospital and ambulatory patients. Although UTIs are not associated with high mortality, they increase the cost of treatment. It is estimated that approximately 70–95% and 5–10% of UTIs are caused by *Escherichia coli* and *K. pneumoniae*, respectively [2]. Nevertheless, according to the World Health Organization (WHO) *K. pneumoniae* is one of the high-priority species due to a growing global problem of antimicrobial resistance [3]. New Delhi metallo-β-lactamase-1 (NDM-1) is the most recently discovered carbapenemase capable of hydrolyzing almost all β-lactams present in Gram-negative pathogens produced mainly by *K. pneumoniae*, and responsible for hospital and acquired infections in community. Uropathogenic bacteria are equipped with special virulence factors that promote colonization of epithelial cells, such as production of adhesins, siderophores, and toxins [4]. In the same way, these bacteria can adhere to medical devices to form biofilm structures. This enables them to avoid immune system responses, thereby rendering antimicrobial therapy unsuccessful.

Since bacteria are becoming constantly more resistant to drugs, more and more researchers all around the world are looking for new and effective methods to combat pathogens and related diseases. Due to antibacterial drugs, we can overcome many infections. However, strains that have developed resistance mechanisms to common antibiotics still pose a significant burden [5]. The idea of using essential oils (EOs) and their compounds (EOCs) to fight bacteria has been increasingly successful, and work on their implementation into treatment has accelerated significantly over the past decade.

Multidirectional activity of EOs and EOCs is widely described in literature: among them antioxidants, antimutagenic, anticarcinogenic, anti-inflammatory, allelopathic, repellent, insecticidal, antiviral, antifungal and antibacterial properties are highlighted. EOs and EOCs are widely used in food, cosmetic, and pharmaceutical industries. Nowadays, they are often found in dietary supplements, herbal medicinal products, syrups, herbs for brewing, and oral liquids [6]. EOs or EOCs have been proved to be able to directly penetrate the bacterial membrane as well as exhibit anti-biofilm effects [7,8,9]. For instance, Kachur and Suntres [10] described that phenolic isomers, carvacrol and thymol, known as very effective antibacterial agents, worked through disruption of the bacterial membrane, which lead to bacterial lysis and leakage of intracellular contents e.g., adenosine triphosphate (ATP). They can also prevent formation of biofilms, inhibit efflux pumps, and bacterial motility. Besides, they may also exhibit additive or synergistic effects in combination with antibiotics.

A very serious problem consisting of the spread of drug-resistant micro-organisms makes us look for new, active compounds that will both have antimicrobial properties and prevent drug resistance. Trifan et al. [11] in their review report presented EOs as ingredients that can be applicable in combinatorial and nano-based strategies in the fight against multi-drug resistant pathogens, also called “ESKAPE” organisms (*Enterococcus* spp., *Staphylococcus aureus*, *Klebsiella* spp., *Acinetobacter baumannii*, *Pseudomonas aeruginosa*, and *Enterobacter* spp.). In our previous study, we analysed the antibacterial activity of selected EOs against extended-spectrum β-lactamase-producing and NDM-1-producing *K. pneumoniae* strains [12]. However, in the current study, we decided to take selected EOCs, including linalool, β-citronellol, linalyl acetate, menthone, (−)-menthol, (+)-menthol, geraniol, eugenol, thymol, *trans*-anethole, farnesol, β-caryophyllene, (R)-(+)-limonene, 1,8-cineole, and carvacrol into consideration. Thus, the aim of this study is to investigate antibacterial and anti-biofilm activities of the above mentioned EOCs against NDM-1-producing uropathogenic *K. pneumoniae* strains.

## 2. Results

### 2.1. Gene Analysis

**Figure 1:**
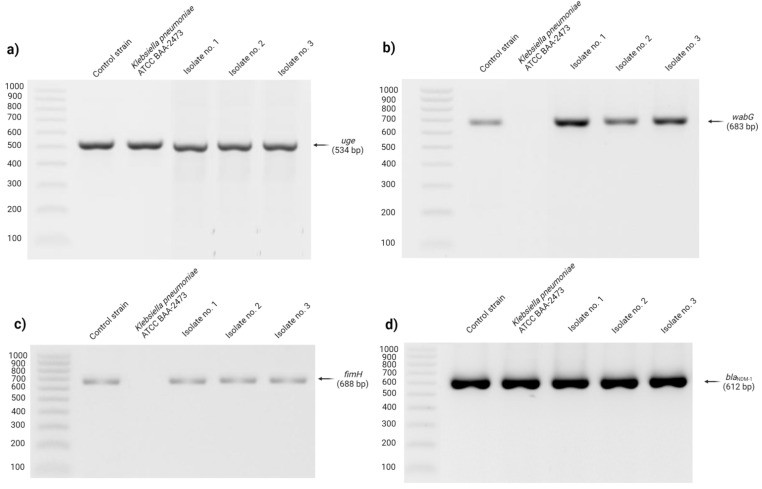
Electrophoresis in 1.5% agarose gel PCR products obtained by using specific primers for *uge* gene (**a**); *wabG* gene (**b**); *fimH* (**c**); and *blaNDM-1* (**d**) gene.

The PCR method enabled detection of all uropathogenic *K. pneumoniae* strains’ genes encoding lipopolysaccharide (*uge*, *wabG*), adhesin gene *fimH* (type I fimbriae) and gene encoding carbapenemase (*bla*_NDM-1_). The *K. pneumoniae* ATCC BAA-2473 reference strain appeared to be *uge*- and *bla*_NDM-1_-positive (Figure 1).

### 2.2. Minimum Inhibitory Concentration (MIC), Minimum Bactericidal Concentration (MBC), MBC/MIC Ratio and the Effectiveness of Investigated Substances against K. pneumoniae Strains

The results showed that the most potent inhibiting activity against all *K. pneumoniae* strains was observed for thymol (MIC: 0.78 ± 0.00 mg/mL; MBC: 1.56 ± 0.00 mg/mL; bactericidal effectiveness). In contrast, the least potent antibacterial activity was observed for menthol (MIC: 224.00 ± 0.00–448.00 ± 0.00 0.0 mg/mL; MBC: >448 mg/mL). Furthermore, it was also shown that all strains were resistant to gentamicin with MIC ranging from 1.25 ± 0.00 to 20.00 ± 0.00 mg/mL. Detailed results of the MICs, MBCs, MIC/MBC ratio and the effectiveness of the investigated substances against *K. pneumoniae* strains are summarized in Table 1. Due to a lack of MBC values for some EOCs and gentamicin, their effectiveness was not determined.

Furthermore, it was also revealed that the Mueller–Hinton broth (MHB) supplemented with 1% (*v*/*v*) Tween 80 or 2% (*v*/*v*) dimethyl sulfoxide (DMSO) did not affect the growth of bacteria.

### 2.3. Effect of Investigated Substances on the Anti-Biofilm Activity

Biofilm biomass reduction assay revealed that in the case of two uropathogenic *K. pneumoniae* isolates (nos. 1 and 2), the use of EOCs and gentamicin at subinhibitory (MIC_50_) concentrations significantly (*p* < 0.0001) decreased biofilm biomass formation. These results appeared to be similar for the reference strain. However, there was no significant statistical difference regarding the effect of MIC_50_ of 1,8-cineole and gentamicin. In turn, the use of investigated substances did not significantly influence formed biofilm biomass in isolate no. 3.

**Figure 2:**
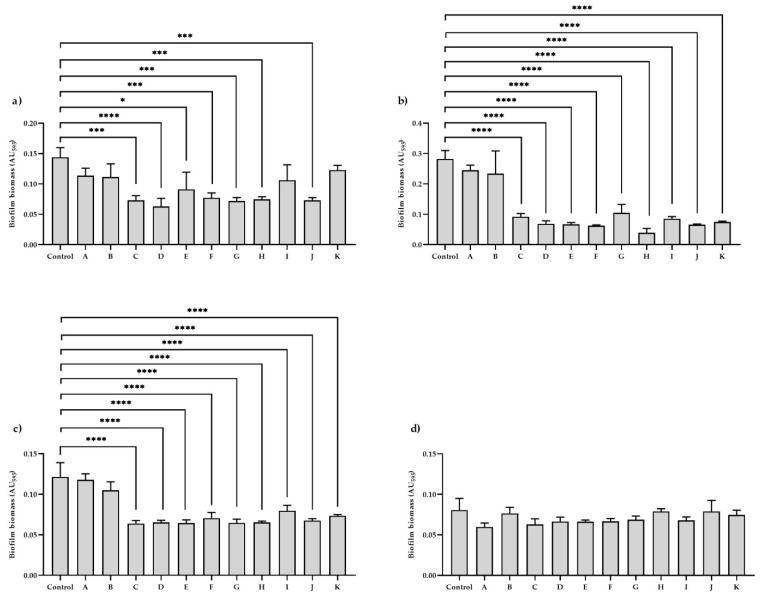
The effect of investigated substances on biofilm biomass reduction by: (**a**) reference strain—*K. pneumoniae* ATCC BAA-2473; (**b**) isolate no. 1; (**c**) isolate no. 2; and (**d**) isolate no. 3. Control—Mueller–Hinton broth (MHB); A—MHB supplemented with 1% (*v*/*v*) Tween 80; B—MHB supplemented with 2% (*v*/*v*) dimethyl sulfoxide (DMSO); C—MHB supplemented with subinhibitory concentration (MIC_50_) of linalool; D—MHB supplemented with MIC_50_ of β-citronellol; E—MHB supplemented with MIC_50_ of menthone; F—MHB supplemented with MIC_50_ of geraniol; G—MHB supplemented with MIC_50_ of eugenol; H—MHB supplemented with MIC_50_ of thymol; I—MHB supplemented with MIC_50_ of 1,8-cineole; J—MHB supplemented with MIC_50_ of carvacrol; K—MHB supplemented with MIC_50_ of gentamicin (positive control). The data are expressed as mean ± standard deviation (SD). Significant differences in biofilm biomass reduction after using different essential oil compounds and gentamicin were considered with the following values: * *p* < 0.05, *** *p* < 0.001, **** *p* < 0.0001.

Moreover, it was found that supplementing the MHB medium with 1% (*v*/*v*) Tween 80 or 2% (*v*/*v*) DMSO did not affect the biofilm biomass reduction. Results of the effect of chemicals on biofilm biomass reduction and a comparative analysis of *p*-values are shown in Figure 2 and Table 2.

## 3. Discussion

It is known that overuse of antibiotics generates various resistant strains, such as NDM-1-producing *K. pneumoniae* strain, which was first detected in 2008 in India in a patient with a urinary tract infection. Safavi et al. [13], in their systematic review, based on data for the years 2010–2019, revealed that the worldwide spread and genotype distribution of human clinical isolates of NDM-producing *K. pneumoniae* observed in Asia, Europe, America, Africa and Oceania was 64.6%, 20.1%, 9.0%, 5.6% and 0.4%, respectively. This type of resistance poses a greater threat since the resistance mechanism, encoded by a gene conditioning the NMD-1 enzyme, has also been detected in other bacterial species, including *E. coli*, *P. aeruginosa*, and *A. baumannii* [14].

Carbapenems were considered one of the most effective groups of drugs for treating bacterial infections. Therefore, growing resistance to these medicaments constitutes a major public health concern. There are not many therapeutic options left after β-lactam and carbapenem antibiotics were withdrawn from use. Treatment alternatives for UTIs are antibiotics such as colistin, Fosfomycin, as well as aminoglycosides, including gentamicin, tobramycin, and amikacin [15]. According to Parente et al. [16], in treatment of pyelonephritis, where Gram-negative bacilli, including multi-resistant *K. pneumoniae*, is the main etiological factor, the paediatric hospitalized population may benefit from an alternative therapy, consisting of a combination of ampicillin and ceftazidime. In the present study, the used strains were resistant to gentamicin, which dramatically reduces therapeutic options of this antibiotic. According to current data of the European Committee on Antimicrobial Susceptibility Testing [17], gentamicin-resistant strains are found at MIC > 2 mg/L. In the current study, MIC for gentamicin against *K. pneumoniae* strains ranged from 1.25 mg/mL (1250 mg/L) to 20 mg/mL (20,000 mg/L).

A carrier of multidrug-resistant *Enterobacteriaceae* and their spread in the hospital environment, which requires implementation of a number of sanitary procedures, also poses a serious problem [18]. That is why it is necessary to search for new agents that could aid treatment, but also would be able to effectively prevent build-up of resistance and spread of these bacteria.

Our current study showed that selected EOCs such as thymol, carvacrol and geraniol exhibited the best antibacterial (showing bactericidal efficacy) and anti-biofilm activities among all tested EOCs against uropathogenic *K. pneumoniae* producing NDM-1. Additionally, these strains harboured the following genes: *uge* (encoding an enzyme involved in envelope synthesis) [19], *wabG* (involved in synthesis of the outer polysaccharide core of lipopolysaccharide) [20], and *fimH* (encoding adhesins) [21]. The *mrkD* gene is dominant in *K. pneumoniae* involved in biofilm formation, but the dominant genes for urinary isolates are also *fimH*, *uge* and *wabG* [22,23]. According to Hamam et al. [23], *fimH* (76%) and *uge* (70%) were the most prevalent genes of biofilm-forming strains of *K. pneumoniae*, isolated from hospital-acquired UTIs. Candan and Aksöz [24] also noted that carbapenems-resistant *K. pneumoniae* isolates, obtained from urine, harboured genes encoding lipopolysaccharide (*uge*, *wabG*), and adhesin gene *fimH* (type I fimbriae). Interestingly, in professional literature, strains harbouring the *wabG* gene have been shown to exhibit higher virulence, which was confirmed especially in UTIs [25].

Among all tested EOCs, thymol (dominant compound of thyme EO) and carvacrol (dominant compound of clove EO) showed bactericidal activity against *K. pneumoniae* NDM-1-producing strains. Thymol and carvacrol, being isomers with similar chemical structures, are likely to demonstrate similar mechanisms of antimicrobial activity but the locations of the hydroxyl groups differ between the two molecules [26]. Antibacterial properties of these compounds are associated with their lipophilic character and their accumulation in cell membranes, which leads to inhibiting electron transport for energy production and disrupting the proton motive force, protein translocation, and synthesis of cellular components. These physiological changes can result in cell lysis and death [27]. Lipopolysaccharide (LPS) in the outer membrane of Gram-negative bacteria is a very drastic barrier for hydrophobic molecules, including hydrophobic antibiotics. Thymol and carvacrol, formed from γ-terpinene, which is lipid in nature, in combination with hydrophobic antibiotics can help to transport them inside the cell [28]. In turn, geraniol was effective against the reference strain and two clinical isolates. Geraniol is an aliphatic monoterpene structure with a functional hydroxyl group. The antimicrobial activity of geraniol is manifested with its ability to adhere to bacterial cell membrane lipids. Geraniol makes the cell membrane more permeable by interacting with its components [29]. Interestingly, in our previous work we found that thyme EO, containing about 38.1% of thymol, had the best antibacterial properties against extended-spectrum β-lactamase (ESBL)-producing and NDM-1-producing *K. pneumoniae* isolates [12]. Interestingly, in the current study, thymol (≥98.5% purity) used alone also exhibited bactericidal properties against reference strain (*K. pneumoniae* ATCC BAA-2473) and all NDM-1-producing uropathogenic *K. pneumoniae* clinical isolates. Our results received for thymol are similar to those obtained by other authors. For example, Bisso Ndezo et al. [30] analysed the effect of thymol against four strains of *K. pneumoniae* isolated from urine. The authors evaluated the MIC and MBC values of thymol against bacteria, which ranged from 0.064–0.256 mg/mL and 0.256–0.512 mg/mL, respectively. Similar results were obtained by Raei et al. [31], who evaluated the effect of thymol and additionally carvacrol on the growth of metallo-β-lactamase-producing *K. pneumoniae* strains. The MIC results for thymol and carvacrol ranged from 0.2 to 1.6 mg/mL and from 0.06 to 0.25 mg/mL, respectively. Moreover, these authors also evaluated the anti-biofilm activity of thymol and carvacrol, which ranged from 0.125 to 0.5 mg/mL and 0.4 to 1.6 mg/mL, respectively. The research conducted during the years 2007–2019 showed that geraniol presents antimicrobial activity against 78 different microorganisms. These results showed that MIC values of geraniol for *K. pneumoniae* were above 1500 µg/mL [29]. This is in line with our results. Yet, we observed a lower MIC value (870 µg/mL) for one isolate. In our study, we also showed significant reduction of biofilm mass of *K. pneumoniae* in the presence of geraniol at sub-inhibitory concentration, compared to biofilm that was non-exposed to geraniol. According to our knowledge, it is the first study concerning the effects of geraniol against biofilm formation by *K. pneumoniae*.

## 4. Materials and Methods

**Figure 3:**
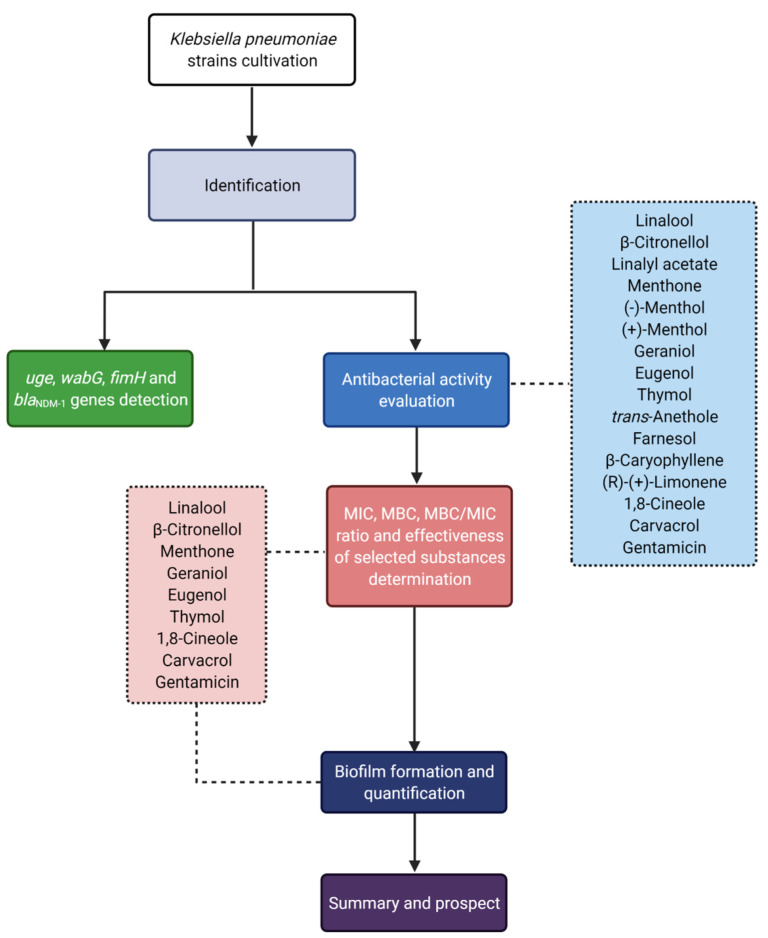
Flow chart of study design.

The flow chart of the experimental design is presented in Figure 3. 

### 4.1. Bacterial Strains and Growth Condition

The study included three NDM-1-producing uropathogenic *K. pneumoniae* strains included in a collection of/collected by the Chair of Microbiology, Immunology and Laboratory Medicine; Pomeranian Medical University in Szczecin (Poland). All the strains were identified using the VITEK 2 Compact system (bioMérieux, Warsaw, Poland), which confirmed their affiliation with *K. pneumoniae* species (≥98%). Before each stage of the experiment the strains had been cultured on Columbia agar with 5% sheep blood (bioMérieux, Warsaw, Poland) and incubated for 24 h at 37 °C under aerobic conditions. The *K. pneumoniae* ATCC BAA-2473 reference strain was used as control.

### 4.2. Investigated Substances

The fifteen EOCs used in this study were: linalool (CAS: 78–70–6; 97% purity), β-citronellol (CAS: 106–22–9; ≥95% purity), linalyl acetate (CAS: 115–95–7; ≥97% purity), menthone (CAS: 10458–14–7; ≥97% purity), (–)-menthol (CAS: 2216–51–5; analytical standard), (+)-menthol (CAS: 15356–60–2; 99% purity), geraniol (CAS: 106–24–1; 98% purity), eugenol (CAS: 97–53–0; 99% purity), thymol (CAS: 89–83–8; ≥98.5% purity), *trans*-anethole (CAS: 4180–23–8; 99% purity), farnesol (CAS: 4602–84–0; 95% purity), β-caryophyllene (CAS: 87–44–5; ≥80% purity), (R)-(+)-limonene (CAS: 5989–27–5; 97% purity), 1,8-cineole (CAS: 470–82–6; primary reference standard), and carvacrol (CAS: 499–75–2; 98% purity) (Table 3).

The EOCs were dissolved in 1% (*v*/*v*) Tween 80 (for linalool, citronellol, linalyl acetate, menthone, geraniol, eugenol, *trans*-anethole, farnesol, β-caryophyllene, (R)-(+)-limonene, 1,8-cineole, and carvacrol) and in 2% (*v*/*v*) DMSO (Loba Chemie, Mumbai, India) (for (–)-menthol, (+)-menthol, and thymol). Using the known densities of EOCs, the results were expressed in mg/mL. The abovementioned chemicals and media were purchased from Merck Life Science (Poznan, Poland). Gentamicin (KRKA, Warszawa, Poland; 40 mg/mL) was used as positive control.

### 4.3. Virulence and Carbapenemase Genes Detection

#### 4.3.1. DNA Isolation

Before the isolation of DNA, the strains were seeded on the Columbia agar with 5% sheep blood and incubated for 24 h at 37 °C. Then, a single bacterial colony was transferred with the use of a bacteriological loop and suspended in 3 mL of tryptic soy broth (Merck Life Science, Poznan, Poland). Next, the suspension was re-incubated for 24 h at 37 °C. Finally, 1.5 mL of the culture suspension was taken for further DNA isolation using GeneMatrix Bacterial & Yeast Genomic DNA Purification Kit (EURx, Gdansk, Poland) according to the manufacturer’s recommendations.

#### 4.3.2. PCR Amplification

PCR amplification was used to detect virulence (*uge*, *wabG*, and *fimH*), and carbapenemase (*bla*_NDM-1_) genes. Primers’ sequences are listed in Table 4. Reference strains, including *E. coli* ATCC 25922, *K. pneumoniae* ATCC BAA-2473 and *K. pneumoniae* ATCC 700603, were used as positive controls.

PCR was conducted using StartWarm HS-PCR Mix (A&A Biotechnology, Gdynia, Poland) mixture. Amplification was conducted using the Applied Biosystems Veriti 96 Well Thermal Cycler (Applied Biosystems, Norwalk, CT, USA) with the following protocol: initial denaturation at 95 °C for four min was followed by 35 cycles of amplification (denaturation—95 °C for 30 s, annealing—53 °C and 52 °C for 30 s respectively for *uge*/*wabG*/*fimH* and *bla*_NDM-1_ genes, extension—72 °C for 60 s) and finished with final extension at 72 °C for 10 min. After PCR, obtained products were analysed by electrophoresis (60 min, 100 V, 1 × tris/borate/ethylenediaminetetraacetic acid) in agarose gel (1.5%, *w*/*v*; DNA Gdansk, Poland) containing 0.5 µg/mL of ethidium bromide (Merck Life Science, Poznan, Poland). PCR products were visualized and photographed using a gel image system (GelDoc-It2 Imager, Analityk Jena US LLC, Upland, CA, USA).

### 4.4. Determination of MIC, MBC, MBC/MIC Ratio and Effectiveness of Investigated Substance against K. pneumoniae Strains

MIC of EOCs and gentamicin against *K. pneumoniae* strains was determined by the serial microdilution method in MHB (Merck Life Science, Poznan, Poland) according to the Clinical and Laboratory Standards Institute recommendations (protocol M07-A9) [36]. Briefly, 50 µL of appropriate concentration of EOCs/gentamicin was added to a 96-well microplate. Then, 50 µL of bacterial suspension at 10^6^ CFU/mL was added to each well of the microplate. After an 18-h incubation at 37 °C, MICs for individual chemicals were determined by adding 20 µL resazurin solution (0.02%, *w*/*v*; Merck Life Science, Poznan, Poland) to the wells [37]. The colour change from dark blue to pink after a 3-h incubation with resazurin at 37 °C indicated the presence of live bacterial cells. The first well in which the dark blue colour persisted determined the MIC value.

MBC (the lowest concentration which kills about 99.9% of bacteria) was determined by transferring 20 µL of bacterial culture at concentrations higher than MIC to a 96-well microplate containing 100 µL of sterile MHB [12]. An incubation was performed for 18 h at 37 °C. After this period, the concentration at which no bacterial growth was observed in the corresponding well was considered as MBC.

To demonstrate the effectiveness of the applied substances the MBC/MIC ratio was calculated [38]. The following ratios: MBC/MIC ≤ 4 and MBC/MIC > 4 were defined as bactericidal and bacteriostatic, respectively.

### 4.5. Effect of Investigated Substances on Biofilm Biomass Reduction

Biofilm biomass reduction was formed according to Barros et al. in sterile 96-wells plates (F-bottom) [39] with a minor modification. Briefly, bacteria were grown onto Columbia agar with 5% sheep blood (Merck Life Science, Poznan, Poland) at 37 °C for 24 h. Then, one colony of each strain was transferred to 3 mL of MHB (Merck Life Science, Poznan, Poland) supplemented with 1% (*w*/*v*) glucose (Merck Life Science, Poznan, Poland) and re-incubated in the same abovementioned condition. Next, 100 µL (5 × 10^5^ CFU/mL) of bacterial suspension was transferred into a microplate with the use of 100 µL subinhibitory concentration (MIC_50_) of EOCs or gentamicin. Simultaneously, control wells were prepared (100 µL of MHB + 100 µL of bacterial suspension). The microplates were incubated at 37 °C for 24 h in static conditions. Subsequently, they were gently washed with phosphate-buffered saline (PBS, pH 7.2) (to remove planktonic cells) and allowed to dry (6 h at room temperature). Then, 4 mL of 0.1% (*w*/*v*) crystal violet (Merck Life Science, Poznan, Poland) solution was added. The samples were left static for 20 min in the dark. The crystal violet solution was removed and the stained biofilms were washed with PBS three times to remove excess unbound dye. Finally, 30% (*v*/*v*) acetic acid (Merck Life Science, Poznan, Poland) solution was added to dissolve the dye and the absorbance values at 595 nm of each well were calculated using a Synergy^TM^ LX Multi-Mode microplate reader (BioTek, Winooski, VT, USA). Acetic acid (30%, *v*/*v*) was used as a blank.

### 4.6. Statistical Analysis

All data were expressed as mean ± standard deviation (SD). All tests were conducted in triplicate. A sstatistical significance between the groups in biofilm biomass reduction assay was measured using the one-way ANOVA test and multiple comparisons. The following values: * *p* < 0.05, ** *p* < 0.01, *** *p* < 0.001, **** *p* < 0.0001 were considered statistically significant. The sstatistical analyses were conducted using GraphPad Prism 8.0.1 (GraphPad Software Inc., San Diego, CA, USA).

## 5. Conclusions

Overall, we can conclude that among investigated EOCs, thymol, carvacrol and geraniol exhibited the best antibacterial and anti-biofilm activities against NDM-1-producing, uropathogenic *K. pneumoniae* isolates. Thus, it seems that these EOCs are promising constituents for development of novel antibacterial combination therapies against biofilm-associated infections. Further studies with the use of more clinical isolates and aiming to investigate the mechanism of action of these combinations are considered. In future, it would be advisable to conduct research on in vivo models of *K. pneumoniae* biofilms. It would be interesting to test a potential application of these compounds in producing active coatings for selected patient care equipment such as catheterization kits.

## Figures and Tables

**Table 1:** Minimum inhibitory concentration (MIC), minimum bactericidal concentration (MBC), MBC/MIC ratio and effectiveness of the investigated substances against *Klebsiella pneumoniae* strains.

Bacteria	Chemicals	MIC (mg/mL)	MBC (mg/mL)	MBC/MIC	Effectiveness
Reference strain (*K. pneumoniae* ATCC BAA-2473)	Linalool	6.48 ± 0.00	51.88 ± 0.00	8	bacteriostatic
	β-Citronellol	6.70 ± 0.00	107.13 ± 0.00	16	bacteriostatic
	Menthone	224.00 ± 0.00	>448	ND	ND
	Geraniol	1.74 ± 0.00	6.95 ± 0.00	4	bactericidal
	Eugenol	4.14 ± 0.00	>530	ND	ND
	Thymol	0.78 ± 0.00	1.56 ± 0.00	2	bactericidal
	1,8-Cineole	57.63 ± 0.00	461.00 ± 0.00	8	bacteriostatic
	Carvacrol	1.91 ± 0.00	1.91 ± 0.00	1	bactericidal
	Gentamicin	1.25 ± 0.00	>40	ND	ND
Isolate no. 1	Linalool	3.24 ± 0.00	25.94 ± 0.00	8	bacteriostatic
	β-Citronellol	26.78 ± 0.00	107.13 ± 0.00	4	bactericidal
	Menthone	448.00 ± 0.00	>448	ND	ND
	Geraniol	3.47 ± 0.00	6.95 ± 0.00	2	bactericidal
	Eugenol	4.14 ± 0.00	>530	ND	ND
	Thymol	0.78 ± 0.00	1.56 ± 0.00	2	bactericidal
	1,8-Cineole	14.41 ± 0.00	461.00 ± 0.00	32	bacteriostatic
	Carvacrol	1.91 ± 0.00	1.91 ± 0.00	1	bactericidal
	Gentamicin	20.00 ± 0.00	40.00 ± 0.00	2	bactericidal
Isolate no. 2	Linalool	1.62 ± 0.00	25.94 ± 0.00	16	bacteriostatic
	β-Citronellol	3.35 ± 0.00	107.13 ± 0.00	32	bacteriostatic
	Menthone	224.00 ± 0.00	>448	ND	ND
	Geraniol	1.74 ± 0.00	6.95 ± 0.00	4	bactericidal
	Eugenol	4.14 ± 0.00	>530	ND	ND
	Thymol	0.78 ± 0.00	1.56 ± 0.00	2	bactericidal
	1,8-Cineole	14.41 ± 0.00	>461	ND	ND
	Carvacrol	1.91 ± 0.00	1.91 ± 0.00	1	bactericidal
	Gentamicin	20.00 ± 0.00	40.00 ± 0.00	2	bactericidal
Isolate no. 3	Linalool	3.24 ± 0.00	103.75 ± 0.00	32	bacteriostatic
	β-Citronellol	1.67 ± 0.00	107.13 ± 0.00	64	bacteriostatic
	Menthone	224.00 ± 0.00	>448	ND	ND
	Geraniol	0.87 ± 0.00	6.95 ± 0.00	8	bacteriostatic
	Eugenol	4.14 ± 0.00	>530	ND	ND
	Thymol	0.78 ± 0.00	1.56 ± 0.00	2	bactericidal
	1,8-Cineole	461.00 ± 0.00	>461	ND	ND
	Carvacrol	1.91 ± 0.00	1.91 ± 0.00	1	bactericidal
	Gentamicin	1.25 ± 0.00	>40	ND	ND

Legend: ND—not determined. Gentamicin was used as positive control.

**Table 2:** Comparative analysis of *p* values obtained in biofilm biomass reduction assay analysed in this study.

Comparison of Group	*p* Value			
	Reference Strain (*K. pneumoniae* ATCC BAA-2473)	Isolate No. 1	Isolate No. 2	Isolate No. 3
Control vs. A	0.413	0.8177	0.9999	0.0652
Control vs. B	0.3198	0.5023	0.2472	0.9999
Control vs. C	0.0003	<0.0001	<0.0001	0.1817
Control vs. D	<0.0001	<0.0001	<0.0001	0.4558
Control vs. E	0.0114	<0.0001	<0.0001	0.4394
Control vs. F	0.0008	<0.0001	<0.0001	0.4683
Control vs. G	0.0003	<0.0001	<0.0001	0.6886
Control vs. H	0.0005	<0.0001	<0.0001	0.9999
Control vs. I	0.1543	<0.0001	<0.0001	0.5959
Control vs. J	0.0004	<0.0001	<0.0001	0.9999
Control vs. K	0.8456	<0.0001	<0.0001	0.9959

Legend: Control—Mueller–Hinton broth (MHB); A—MHB supplemented with 1% (*v*/*v*) Tween 80; B—MHB supplemented with 2% (*v*/*v*) dimethyl sulfoxide (DMSO); C—MHB supplemented with subinhibitory concentration (MIC_50_) of linalool; D—MHB supplemented with MIC_50_ of β-citronellol; E—MHB supplemented with MIC_50_ of menthone; F—MHB supplemented with MIC_50_ of geraniol; G—MHB supplemented with MIC_50_ of eugenol; H—MHB supplemented with MIC_50_ of thymol; I—MHB supplemented with MIC_50_ of 1,8-cineole; J—MHB supplemented with MIC_50_ of carvacrol; K—MHB supplemented with MIC_50_ of gentamicin (positive control).

**Table 3:** Characteristics of the investigated substances (https://www.ncbi.nlm.nih.gov/pccompound) (accessed on 15 December 2021).

Chemicals	Structure	Molecular Formula	Flavor Profile	Application
Linalool	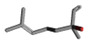	C_10_H_18_O	Coriander, floral, lavender, lemon, rose	Flavouring agent or adjuvant
β-Citronellol	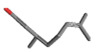	C_10_H_20_O	Citrus, green, rose	Food improvement agent
Linalyl acetate	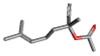	C_12_H_20_O_2_	Fruit	Flavouring agent or adjuvant
Menthone	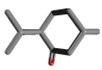	C_10_H_18_O	Green, fresh, mint	Flavouring agent or adjuvant
(−)-Menthol	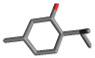	C_10_H_20_O	Mint, Cool	Flavouring agent or adjuvant
(+)-Menthol	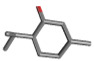	C_10_H_20_O	Mint, Cool	Flavouring agent or adjuvant
Geraniol	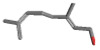	C_10_H_18_O	Geranium, lemon peel, passion fruit, peach, rose	Flavouring agent or adjuvant
Eugenol	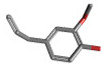	C_10_H_12_O_2_	Burnt, clove, spice	Flavouring agent or adjuvant
Thymol	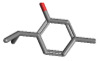	C_10_H_14_O	Spice, wood	Flavouring agent or adjuvant
*trans*-Anethole	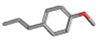	C_10_H_12_O	Anise	Flavouring agent or adjuvant
Farnesol	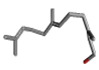	C_15_H_26_O	Oil	Flavouring agent or adjuvant
β-Caryophyllene	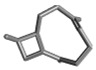	C_15_H_24_	Fried, Spice, Wood	Flavouring agent or adjuvant
(R)-(+)-Limonene	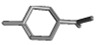	C_10_H_16_	Citrus, Mint	Flavouring agent or adjuvant
1,8-Cineole	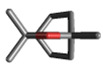	C_10_H_18_O	Camphor, cool, eucalyptol, mint	Flavouring agent or adjuvant
Carvacrol	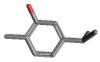	C_10_H_14_O	Caraway, spice, thyme	Flavouring agent or adjuvant

**Table 4:** Primer sequences used in this study.

Gene	Sequence	Amplicon Length (bp)	References
*uge*	F: 5′-GAT CAT CCG GTC TCC CTG TA-3′ R: 5′-TCT TCA CGC CTT CCT TCA CT-3′	534	[32]
*wabG*	F: 5′-CGG ACT GGC AGA TCC ATA TC-3′ R: 5′-ACC ATC GGC CAT TTG ATA GA-3′	683	[33]
*fimH*	F: 5′-ATG AAC GCC TGG TCC TTT GC-3′ R: 5′-GCT GAA CGC CTA TCC CCT GC-3′	688	[34]
*bla*_NDM-1_	F: 5′-GGA ATA GAG TGC CTT AAY TCT C-3′ R: 5′-CGG AAT GGC TCA CGA TC-3′	612	[35]

## Data Availability

The data presented in this study are available in the article.
